# Transfusion-transmitted occult hepatitis B virus infection: current understanding, challenges, and its implication in blood safety

**DOI:** 10.3389/fimmu.2025.1663306

**Published:** 2025-10-28

**Authors:** Linbin Huang, Baoren He, Qiuhong Mo, Bin Li, Xipeng Yan, Rongji Lai, Xinwei Wang, Jinlian Li, Mingshuang Lai, He Xie, Jujun Sun, Xianlin Ye, Limin Chen

**Affiliations:** ^1^ The Joint Laboratory on Transfusion-Transmitted Diseases (TTDs) Between Institute of Blood Transfusion, Chinese Academy of Medical Sciences and Nanning Blood Center, Nanning Blood Center, Nanning, China; ^2^ The Hospital of Xidian Group Laboratory Department, Xi’an, China; ^3^ Shenzhen Blood Center Laboratory Department, Shenzhen, China; ^4^ Institute of Blood Transfusion, Chinese Academy of Medical Sciences and Peking Union Medical College, Chengdu, China

**Keywords:** occult hepatitis B virus infection (OBI), blood transfusion transmission, blood testing, blood safety, HBV prevention and control

## Abstract

Occult hepatitis B virus infection (OBI) represents a specific form of hepatitis B virus (HBV)infection characterized by the presence of replication-competent HBV DNA in the liver despite a negative blood test for hepatitis B surface antigen (HBsAg). Due to the incompletely-known mechanisms underlying its occurrence and the limitations of existing screening technologies, the viral loads in the blood of OBI patients are intermittent and often undetectable. Furthermore, lack of effective screening and shielding strategies in blood collection and supply institutions fail to prevent OBI individuals from donating blood, resulting in its susceptibility to transmission through blood transfusion, which poses a significant threat to blood safety. In this review, we summarize current understanding of OBI, challenges, and its implication in blood safety.

## 1 Introduction

HBV infection causes a significant liver disease and poses a global public health challenge. The World Health Organization (WHO) estimates that approximately 296 million people worldwide were chronically infected with HBV in 2019, resulting in 820,000 deaths, primarily due to cirrhosis and hepatocellular carcinoma (HCC) (1, 2). Although WHO has established a mandate to eliminate HBV by 2030 (2, 3), OBI remains difficult to be detected in routine blood screening due to its low viral load and intermittent nature. Previous study indicates that transfusions from donors with OBI are estimated to cause HBV infection in 8-29% of recipients (4), thereby presenting a significant threat to blood safety. We summarize the pathogenesis and prognosis of OBI, its epidemiology, the limitations of current diagnostic and screening techniques, the impact on blood safety, and strategies for its prevention and control in order to ensure blood safety.

## 2 The definition of OBI

Transfusion safety issues have been an increasing concern among researchers since the late 1970s, when studies confirmed that blood from donors containing hepatitis B core antibodies (anti-HBc) but no detectable HBsAg or hepatitis B surface antibodies (anti-HBs) could transmit hepatitis B through blood transfusions (5, 6). Subsequently, with the development of molecular biology techniques, cases of HBsAg-negative but HBV DNA-positive liver disease and transmission were discovered, which gradually led to the definition of “occult hepatitis B infection (OBI)” (7–12).

OBI is defined as the presence of replication-competent HBV DNA (*i.e.* episomal HBV covalently closed circular DNA [cccDNA]) in the liver and/or HBV DNA in the blood of people who test negative for HBsAg by currently available assays (13, 14). Due to strong suppression and clearance pressure from the host immune system, HBV cccDNA exhibits low-level replication typically below 200 IU/mL or intermittent occurrence, which pose significant challenges to routine screening assays (13–16). Specifically, currently available OBI screening technologies frequently fail to detect OBI due to insufficient sensitivity or inadequate ability to detect virus mutations (17). Improvements in quantitative HBV marker detection methods and enhancements in the performance of detection reagents and instruments can significantly increase the OBI yields or reclassify them as chronic HBV infections (CHB) (18–22).

**Figure 1:**
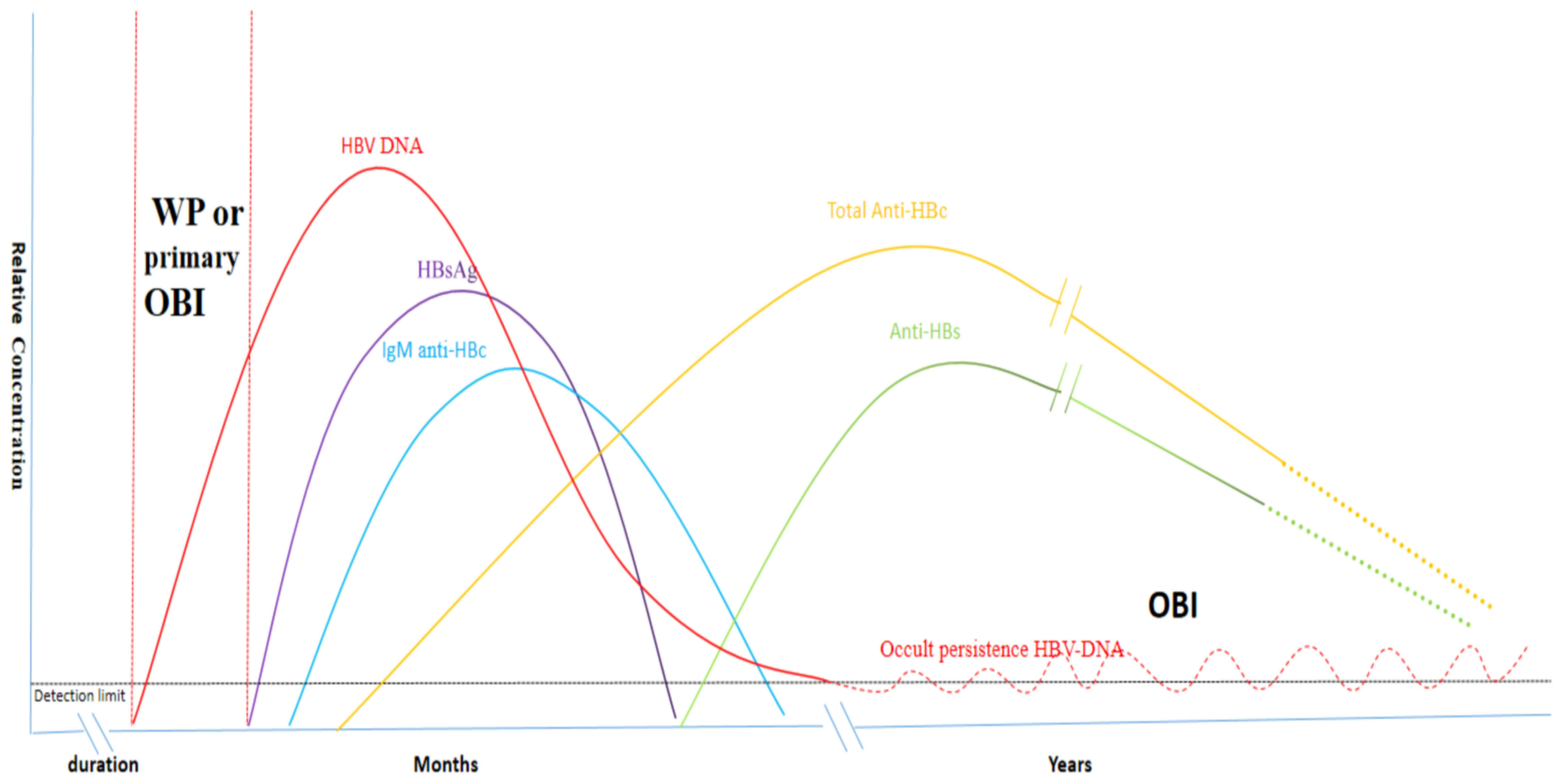
Serological markers following HBV infection and the occurrence of OBIs (2, 28) Specific serological markers, including HBV DNA, HBsAg, anti-HBc, and anti-HBs are detected in the blood following HBV infection. OBIs primarily arise from the virus’s low replication state in response to host immune pressure. In this condition, HBV DNA levels fluctuate around the lowest detection limit and are only intermittently detectable. In some cases, anti-HBc and anti-HBs gradually decline and HBV DNA is the only detectable marker.

Accurate identification of “occult” and “overt” HBV infection status is valuable for HBV prevention and control. In routine testing, serological testing is primarily used to accurately identify these two infection states. Two types of serological test results are often presented in OBIs: those that are positive(80%) for anti-HBc and/or anti-HBs, and those that are negative(20%) for both (13, 14, 23, 24). Especially, due to the lack of serological markers, serological negative OBI and the window phase (WP) of acute or chronic hepatitis B infection are classified as “primary OBI” (1, 13, 25, 26). In contrast, “overt” HBV infection is defined as the presence of HBsAg and viral genomic DNA in the blood, which indicates abundant replication and high transcriptional activity of the virus in the host (1, 27). As shown in **Figure 1**, HBV infects the host and gradually produces specific markers such as, HBV DNA, HBsAg, anti-HBc, and anti-HBs as the virus continues to replicate. However, when encountering strong immune pressure from the host, HBV exists in a state of low replication state in which HBV DNA levels fluctuate around the lowest detection limit and are only detected intermittently. This is commonly denoted as the OBI state, and in some cases, anti-HBc and anti-HBs gradually disappear, while HBV DNA is the only detectable marker.

## 3 Mechanisms of OBI formation

OBI results from a complex interplay between HBV and the host. HBV utilizes the host receptor to gain entry into hepatocytes, where rcDNA is released and subsequently converted to cccDNA. This cccDNA serves as a template for the generation of pgRNA and mRNA, ultimately leading to the transcription of HBV DNA and the production of various viral proteins (1). However, due to host immune response, epigenetic regulation, and viral mutation, HBV cccDNA may exist in a low replication state leading to very low level of HBV DNA without detectable HBsAg in the blood and/or liver tissue, and OBI arises (14, 29, 30).

Although the detailed mechanism of OBI formation is not completely understood, many studies indicate that host immune response, viral escape mutation, epigenetic regulation and co-infection with other viruses may play an important role.

Firstly, the role of host immune response to HBV infection in OBI occurrence should not be neglected. Under strong immune pressure, HBV replication is significantly suppressed leading to low viral loads and undetectable HBsAg (31, 32).

Secondly, the appearance of viral escape mutations is also important. HBV genome contains four open reading frames (S,X,P,C), and in order to be able to adapt to the host environment, the virus undergoes continuous mutations in order to escape the immune surveillance (33–35). Some mutations in the HBsAg “α” determinant cluster (36, 37), S-region pres1/pres2 promoter (18, 33, 38), BCP/PC region (38), and the HBsAg hydrophilic region (MHR) (35) affect not only the structure but also the production/secretion of HBsAg, which favor viral immune evasion and ultimately promote OBI formation.

Thirdly, epigenetic regulation may also involve in the formation of OBIs. HBV cccDNA is similar to the chromatin of host cells and histone modifications by methylases and acetylases have been shown to inhibit viral replication (39–41). In addition, previous study reported a natural glycosylation pattern of HBsAg, which may escape immune-mediated clearance by masking antigenic determinants, leading to the development of cryptic infections (42).

**Figure 2:**
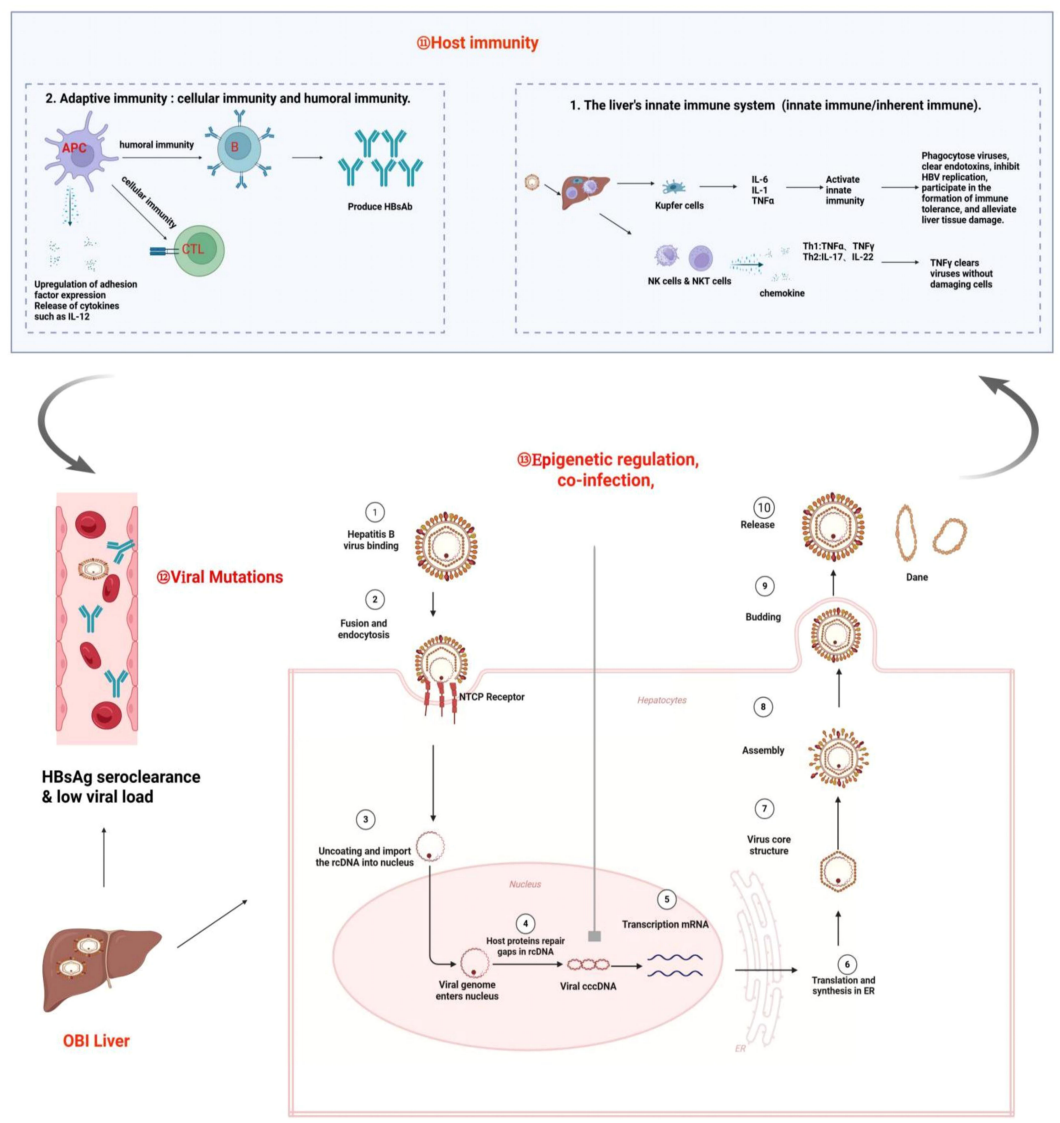
Mechanisms of OBI formation. OBI is the result of complex interactions between the HBV and the host. Under the influence of various factors, such as strong immune suppression and clearance by the host, epigenetic regulation, co-infection, and mutation, the OBI state is ultimately formed, which is characterized by the presence of replication-competent viral DNA in the liver (detectable or undetectable HBV DNA in serum), while the individual tests for HBsAg are negative.

Last but not the least, co-infection of HBV with other viruses such as HIV or HCV has been shown to inhibit HBV replication and decrease HBsAg production/secretion leading to OBI (43, 44). **Figure 2** briefly summarizes the potential mechanisms of OBI formation following HBV infection.

As for the outcomes of OBIs, retrospective studies from OBI blood donors have shown that few OBI-infected patients spontaneously clear HBV from their bodies (45, 46), whereas the majority of them ultimately remain in a state of low viral replication and intermittently detectable HBV DNA (18, 47). As such, transfusion-transmitted HBV infection may occur if blood collection and supply institutions do not shield these OBI donors.

## 4 Challenges in OBI diagnosis and screening in blood institutions

**Figure 3:**
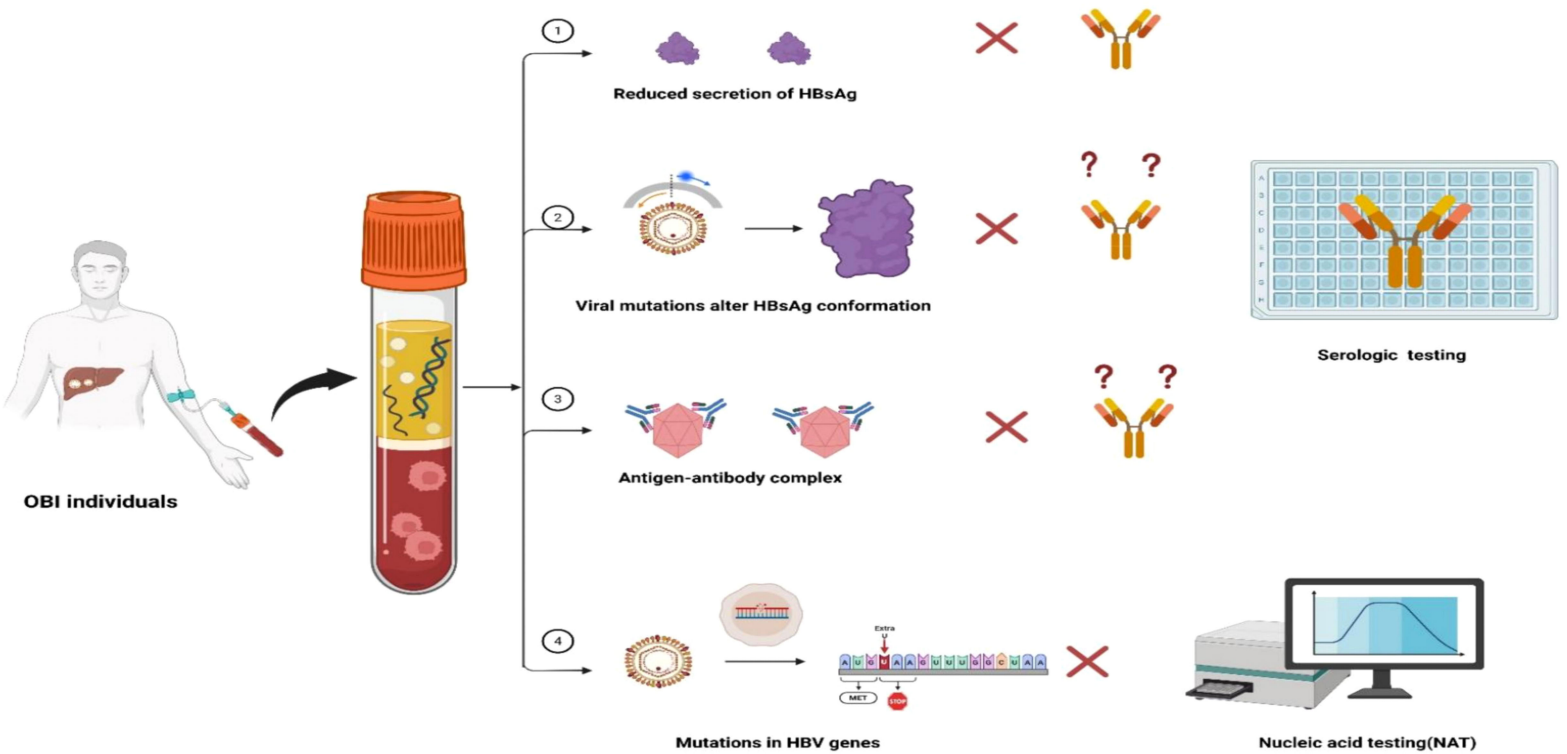
Challenges in OBI diagnosis and screening. ① Reduced secretion of HBsAg caused by Host immunity pressure suppressed the secretion of HBsAg, which is under detection limit of the commercial reagents leading to detection failure. ② Viral mutations cause changes in the spatial conformation of HBsAg, which cannot be recognized by monoclonal antibodies and lead to detection failure of commercial reagents. ③ Formation of antigen-antibody complexes in the blood, which cannot be recognized by commercial reagents and lead to detection failure. ④ Mutations in viral genes may result in altered gene sequences or reduced synthesis of HBV DNA, which then lead to testing failure by NAT.

The diagnosis and screening of OBI face several challenges. Due to the low viral load and intermittent viremia inherently associated with OBIs, the viral load may be under the detectable level, resulting in missed diagnoses (18, 48, 49). Although the detection of cccDNA in the liver tissue is considered the golden standard for the diagnosis of OBI, liver biopsy process itself is invasive, and there is no standardized techniques for detecting HBV DNA in liver tissues (13, 14). Therefore, screening for OBI primarily relies on serological and molecular testing techniques. However, in practice, several factors contribute to the underdiagnosis of OBIs, including the limited sensitivity of testing methods (50, 51), inability to detect viral variants (52), inappropriate ratios of anti-HBs to HBsAg in the samples (21), and the variability in testing modes and algorithms (53). Challenges in OBI screening are summarized in **Figure 3**.

### 4.1 Challenges in serologic testing

HBsAg is widely used as a serum marker for screening and diagnosis of HBV infection, and the lowest limit of detection of the commercial reagents is 0.05 IU/mL (54, 55). Some studies demonstrated that 18.2%–61.5% of samples previously classified as HBsAg negative by conventional assays were tested positive for HBsAg by ultrasensitive methods (17). In addition, the insufficient ability of commercial testing reagents to detect virus mutations is another factor contributing to missed detection of OBIs. Once the altered spatial conformation or reduced secretion of the HBsAg derived from HBV mutations occur, HBsAg cannot be recognized by the commercial monoclonal antibody reagent, resulting in testing failures (52, 56). In addition, the formation of antigen-antibody complexes in OBI blood can also cause detection failure (21, 34, 57).

### 4.2 Challenges in nucleic acid testing

Nucleic acid testing (NAT) used for blood screening primarily include transcription-mediated nucleic acid amplification assays (TMA) and real-time polymerase chain reaction (PCR) (47). For NAT, the lower limit of most commercially available HBV DNA tests ranges from 10 to 20 IU/ml, while for blood products sensitivity is higher (1.4 to 12 IU/ml) and specificity (99.9%) in blood-supplying institutions (53, 58, 59).The sensitivity of the mini pool (MP-NAT) decreases as the number of samples mixed in the mini pool increases, suggesting that insufficient sensitivity of the MP-NAT testing could also lead to missed OBIs detection (60, 61). Additionally, an American comprehensive study from 22.4 million blood donors revealed that only 43/404 (10.6%) OBIs could be detected by MP-NAT, and most OBIs (361/404, 89.4%) could only be identified by individual testing (ID-NAT) (19). In addition, when the viral genome mutates, it cannot be amplified by primer recognition, resulting in nucleic acid detection failure (62, 63). Notably, given that the HBV DNA level of OBI usually fluctuates around the lowest detection limit, and is only detectable intermittently, these factors also increased the possibility of testing failure (61). Further, although the laboratory’s MPX Taq screening assay is a quantitative method in which the Ct value reflects the viral load in the blood sample, it follows Poisson distribution and the Ct value may fluctuate when the viral load is close to the lowest detection limit, leading to inconsistent results and undetectable OBIs (53, 61, 64).

## 5 The prevalence of OBIs in blood donors

The global prevalence of OBIs varies significantly due to differences in geographical locations, detection sensitivities of assay used, host immune responses, and vaccine availability, and it ranges from 0.06%-12% in general population (4). As shown in **Table 1**, meta-analyses indicate the prevalence of OBIs in blood donors ranges from 0.0003%-16.48% depending on different study populations. Even in the same country (E.g., China), the prevalence varies considerably in different regions, and prevalence of OBIs in blood donors in Heyuan is 0.16% while 0.0378% in Zhejiang.

**Table 1:** The prevalence of OBIs in blood donors in different countries/regions/city.

Country/region/city	Year	OBI yield (%)	Reference
Sudan	2020	16.48	(76)
Guinea	2022	15.60	(77)
Egypt	2023	12.00	(78)
Cameroon	2024	4.5	(37)
Africa	2024	3.18	(70)
Vietnam	2022	0.30	(79)
He Yuan, China	2022	0.16	(18)
Da Lian, China	2022	0.097	(46)
Switzerland	2022	0.061	(65)
Zhe Jiang, China	2021	0.0378	(47)
France	2022	0.0071	(66)
Madrid, Spain	2020	0.003	(80)
England	2021	0.00038	(67)
The United States	2018	0.0018	(19)
Australia	2019	0.0013	(81)
Canada	2019	0.0003	(81)

It is important to note that the prevalence of OBIs may be underestimated due to several factors: a) Sample limitations: many OBI-related studies rely on limited retrospective data and the OBI-containing blood/organs may have been used clinically even when OBIs were identified (65–67). b) Lack of screening and shielding policies: Nucleic acid testing has not been routinely performed in low- and middle-income countries, leading to many cases of undetected OBIs (37, 68–73). Furthermore, most countries have no shielding policies in place for OBI donors, which results in blood donations from repeated OBI donors, increasing the risk of transfusion-transmitted HBV infection (72, 74). c) Inadequate sample storage: The absence of donor and/or recipient samples complicates traceability (67). And d) Differences in analytical methods: Variations in the analytical methods employed across different studies affect the consistency of the results (16, 67, 75).

## 6 The impact of OBIs on blood safety

It is extremely difficult to detect OBIs by routine screening assays, which may result in HBV infections in recipients (49, 82). Previous study indicated that HBV infection was detected in 8-29% (4) of recipients who received OBI-containing blood. Moreover, the absence of effective screening and shielding policies for OBI donors increased TT-OBIs (74, 75). It is worth noting that a single bag of donated blood may be processed to different blood products. In other words, a single bag of OBI blood may lead to HBV infection in multiple recipients (49). Unfortunately, most studies investigating OBI transmissions lack experimental validation and clinicians may overlook patients who do not exhibit overt signs of acute hepatitis (13, 28). Furthermore, healthcare workers may be hesitant to trace infections to avoid potential medical disputes, missing opportunities for timely intervention following transfusion of OBI blood (49).

After being transfused with OBI blood, recipients may be infected with HBV and this leads to clinical transfusion safety incidents (83–86). For instance, A recent study showed that anti-HBc positivity increased to 37.7% in children with leukemia who received blood transfusions during immunosuppressive therapy, suggesting that transfusion of potentially OBI-containing blood may lead to passive transfer of anti-HBc and anti-HBs (87). Likewise, a study by Allain et al. (84) showed that 3 patients developed sepsis and hepatitis after receiving blood products containing OBI. In the worst scenario, Spreafico et al. (83) reported that a case of a bone marrow transplant patient with a hematologic disorder who received OBI blood resulted in viral activation and eventual death from acute liver failure. Examples of TT-OBIs in different countries/regions were summarized in **Table 2**.

**Table 2:** TT-OBIs reported in different countries/regions.

Year	Country/region	Number of OBI blood (units)	Ratio of recipients infected (%)	HBV Genotype	Reference
2007	Japan	95	1/33 (3)	C (A)	(88)
2011	HongKong, China	10(67)	1/49 (2)	B/C	(89)
2011	Taiwan, China	11(75)	3/13 (23)	B	(90)
2013	Europe	24 (105)	46/105-15(29)	A/D	(84)
2015	Australia	/	(0.2–3. 3)	/	(91)
2019	UK	3(30)	9/31(29)	D	(49)
2015	Italy	14	2/14(18.3)	D	(83)
2016	Netherlands	16	4/82(5)	/	(92)
2021	England	25	25/655(3.8)	D/A/E	(67)

## 7 Strategies to reduce the risk of TT-OBIs to enhance blood safety

### 7.1 HBV vaccination

Vaccine administration is one of the most cost-effective measures to control infectious diseases. For example, China introduced the routine immunization with hepatitis B vaccine in 1992 and this significantly reduced the prevalence of HBsAg to 1% in children under five years of age (93). However, the levels of protective antibodies induced by the vaccine can gradually wane over time, resulting in diminished protection and an increased risk of breakthrough infections (94–96).Therefore, regular surveillance and identification of breakthrough infections following hepatitis B vaccinations are essential for the long-term prevention of HBV infection (97). This is particularly important for some special groups such as blood donors, those with hepatitis B-positive family members, public service workers, and medical waste handlers (98–100). Consequently, regular testing for hepatitis B markers and boost vaccination if needed should be strongly encouraged (74, 101, 102).

### 7.2 Selection of blood donors

Despite significant advances in laboratory screening technologies, the initial step in ensuring blood safety is the selection of unpaid voluntary blood donors who are at lower risk (60). HBV can be transmitted through various routes, including vertical transmission, blood transfusion, intravenous drug use, sexual contact, tattoos, and piercings (100, 103, 104). Blood donations from individuals with “high-risk behaviors” can be effectively excluded through rigorous counseling and screening, which represents an economically feasible and practical approach to ensure blood safety (72, 77, 103, 105).

### 7.3 Pathogen inactivation procedures

Pathogen inactivation (PI) represents a strategic approach to mitigate the risks associated with transfusion-transmitted infections and has been successfully implemented in numerous countries worldwide. It is recommended that all labile blood products and biologics undergo pathogen inactivation to ensure blood safety and sustain a secure blood supply (106).

### 7.4 Novel testing techniques and biomarkers

Given the low viral load and intermittent viremia characteristics of OBI, it is imperative to improve sensitivity and the ability to identify viral variants of the screening assays (63). This can be achieved by optimizing the detection of various open reading frames in the genome of HBV and multiple epitopes of the viral proteins (13, 24), as well as by incorporating more sensitive nucleic acid detection methods, such as real-time PCR, nested PCR, and digital PCR (14). Additionally, considering the intermittent appearance of HBV DNA in the blood, blood specimens should be collected at multiple time points. This can be combined with ultra-high-speed centrifugation, specific adsorption, and other techniques to concentrate the virus before assay was performed (48, 65), or by increasing the volume of plasma or serum used for DNA extraction (48, 63).

Importantly, the limitations of serum HBVDNA testing underscore the necessity for complementary testing strategies based on other biomarkers from HBV infection. Anti-HBc testing should be implemented, particularly when obtaining liver tissue is challenging or when HBV DNA is negative but the possibility of OBI cannot be excluded (107, 108). For instance, a study by Ye et al. (107) indicated that among 103,356 seronegative blood donors, there were 252 non-resolved donations (which had been released for clinical use). In these 252 cases, alternative methods combining NAT with Nested PCR + qPCR and Roche ECLI were used for serological testing (HBsAg/anti-HBs/anti-HBc/HBeAg/anti-HBe). Ultimately, 17 cases were identified as HBVDNA positive (with all anti-HBc being positive). Previous studies reported that anti-HBc testing was effective in identifying blood donors with OBIs (2, 14). That is, anti-HBc screening combined with high-sensitivity HBV NAT screening can effectively prevent almost all HBV transmission from OBI donors (107, 109). In Japan (110) and France (66), for example, the implementation of anti-HBc screening has significantly reduced the incidence of transfusion-transmitted HBV infections. However, anti-HBc testing for OBIs also has limitations. The sensitivity and specificity of anti-HBc in identifying occult HBV infections are only 77% and 76%, respectively (4). Moreover, anti-HBc screening may be impractical in HBV high prevalent countries, such as in China, where anti-HBc screening would eliminate at least 36% of eligible blood donors (111). Therefore, blood services in each country should decide whether or not to implement anti-HBc testing in the context of HBV prevalence (112).

Notably, this rare problem should not be overlooked, as we currently know very little about the clinical significance of anti-HBc-negative OBI and serological negative OBI donors (109). In particular, low levels of HBV DNA and intermittent testing can easily lead to false-negative HBV DNA test results. If we rely solely on serological testing, it is easy to miss some of these serological negative OBI cases (95). Studies have shown that a small number of OBI are HBVDNA-negative and have all serological markers negative (48, 52). In addition, it is worth mentioning that donors with only anti-HBs-positive serological characteristics may be in the acute WP or OBI, which has been reported to account for 4.4% (26) to 11% (113) of cases and has been associated with blood transfusion-transmitted infection (109, 114). Similarly, the use of anti-HBs testing should also be evaluated on a case-by-case basis (114). In practice, for example, in order to prevent serious blood shortages caused by excluding only anti-HBc-positive individuals, the Japanese Red Cross(JRC) Blood Center took measures in 1989 to accept blood from donors with elevated anti-HBs levels(≥200IU/ml) and low anti-HBc levels. However, transfusion-transmitted HBV infection cases still occurred (88, 115). Further, it should be noted that although high levels of anti-HBs may reduce the risk of HBV infection to a certain extent (84, 115), they may not provide sufficient protection for immunodeficient recipients (46, 47, 82).

Since its introduction in the 1970s, OBIs have remained an area of active investigation. With the advancement of new technologies, a variety of markers and screening techniques have been emerging in clinical practice over the past two decades, and these HBV- related biomarkers include serum HBV RNA, hepatitis B core-related antigen (HBcrAg), quantitative HBsAg (qHBsAg), quantitative anti-HBc (qAHBc), and HBV nucleic acid- related antigen (HBV-NRAg) (116–118). Some immunological techniques have made great breakthroughs, such as Lumipulse HBsAg Hypersensitivity Assay, the sensitivity, specificity and coefficient of variation (CV) reaches 0.005 IU/mL, 99.8% and <4%, respectively (51). More valuably, its performance is not affected by clinical treatments, viral gene mutations, or antigen-antibody complexes, establishing a foundation for early diagnosis and treatment to facilitate the mechanistic study of the OBI formation (17, 51, 119). Molecular diagnostic technologies, particularly those based on Clustered Regularly Interspaced Short Palindromic Repeats (CRISPR), provide new approaches for rapid, sensitive, and portable nucleic acid molecular detection (120, 121). The detection sensitivity is as low as 0.05 ng/mL, with 100% specificity (122),which was successfully applied to the detection of HBV DNA with low viral load (123)and HBV cccDNA (124). Next Generation Sequencing (NGS) serves as a powerful tool for the detection and research of HBV (125, 126), which is a robust technology that detects integrated HBV DNA (iDNA) in blood and urine even in CHB patients with a sensitivity of serum viral load <2 IU/mL or even undetectable, and a specificity of at least 99.9% (127). Particularly in the identification and mechanistic exploration of OBIs. Despite notable progress in sensitivity and specificity of detection methods, challenges persist for low-level HBV markers typical of OBIs, particularly regarding result stability and reproducibility (47, 53, 61, 64, 128). Transfusion-related cases repeatedly showed that several assays underperformed at low viral loads, raising the risk of OBIs going undetected (49, 84). Some other investigators have proposed that the minimum infectious dose of HBV in blood transfusion may be as low as 16copies/ml or 3 IU (49). In order to convey these information, we added **Table 3** to summarize the performance of currently commercialized quantitative HBV markers.

**Table 3:** The performance of currently commercialized quantitative HBV markers.

HBV markers	Detection technology	Sensitivity LOD, IU/ml	Specificity	Coefficient of variation (CV)	Repeatability	Note
HBsAg	ELISA (56)	0.05 (WHO standard 0.011-0.095)	NA	NA	NA	
	Architect HBsAg Next (20, 21, 131)	0.005	99.95-100%	<6%	NA	HBsAgNx improved OBI yield by 5% (20)–22.3% (21).
	Elecsys HBsAg II(Cobas e 601/e602) (132)	0.05	100%	<3.2%	NA	HISCL HBsAg and Elecsys HBsAg II quantitative detection and confirmation results had consistency rates of 98.13% and 96.79%, respectively. HBsAg ELISA test result was negative, but ECLI 0.77% was positive (titers were 0.11 IU/L and 1.73 IU/L, respectively) (52).
	HISCL HBsAg (133)	0.03	NA	< 15%	NA	
Anti-HBs	Anti-HBs(Elecsys anti-HBs assay (Roche Diagnostics) (20, 52)	2	NA	NA	NA	➢ Anti-HBs levels above 300 mIU/mL may interfere with the detection of samples with extremely low viral loads (21). ➢ Anti-HBs has a relatively limited lifespan in circulation, but it can be detected in OBI individuals with HBV-DNA and anti-HBc (134). ➢ Enhanced anti-HBs levels promote the body’s immune clearance of HBV, reducing the risk of HBV-related cirrhosis and HCC (97). ➢ Low viral load seems to be inversely proportional to anti-HBs levels and directly proportional to anti-HBc levels (47, 52).
	Architect anti-HBs assay(Abbott Laboratories) (20)	10	NA	NA	NA	
qHBeAg	Roche Diagnostics’ Elecsys platform (135)	0.30	NA	NA	NA	Anti-HBe often coexist with anti-HBc, which is an indicator that virus replication is under control (136).
qAnti-HBc	HBcAb-HS (137)	0.005 - 1.500	100%	NA	NA	Anti-HBc is the earliest specific antibody to HBV infection and remains detectable throughout chronic infection (88). It can be combined with highly sensitive NAT for the diagnosis of OBI (107, 109). The combination between qAnti-HBc and qHBsAg provided a significant predictive value for HBsAg clearance (138).
HBcrAg	LUMIPULSE G HBcrAg assay (Fujirebio, Tokyo, Japan) (139)	3 log U/ml	NA	NA	NA	In the 23 OBI samples with low HBV DNA levels (mean 29.7 IU/mL), 20 had HBcrAg levels below the detection limit, and two were close to the lower limit of the HBcrAg measurement range (21).
HBV pgRNA	Abbott m2000sp/rt system (140)	1.65 log U/ml	NA	NA	NA	In 23 OBI samples with low HBV DNA levels (mean 29.7 IU/mL), only 2 were pgRNA-positive (21).
q HBVRNA (qpgRNA)	dual-target(ORF-X& ORF-C) qRT-PCR approach on the Abbott m2000sp/rt system (140)	ORF-X LOD: 44.7 ORF-C LOD: 46.8 ORF-X LOQ: 44.7 ORF-C LOQ: 64.6	100%	NA	NA	HBV DNA viral loads ranged from undetectable to 8.6 log IU/ml. HBV pgRNA was detected in all samples (140).
NRAg (HBsAg/PreS1Ag)	Hybrid Capture system from Murex3(Abbott Diagnostics, Rungis, France) (141)	50 pg/ml	NA	NA	Good intraassay reproducibility with a standard deviation of <1%.	
HBV DNA	RT-PCR assay on Roche cobas 4800 System (142)	LOD:4.4 LOQ:10.0	100%	0.5% - 3.5%	LOD: 91% LOQ: 37.5% LOB: 100%	Limit of detection (LOD) 4.4 IU/ml limit of quantitation (LOQ) 10.0 IU/ml limit of blank (LOB)
	Roche cobas 6800 (143, 144)	1.48	100%	≤15%	NA	
	Cobas TaqScreen MPX test version 2.0 (Roche Molecular Systems, Inc., NJ, USA) (17)	2.3	NA	NA	NA	
	Quantitative HBV DNA(Roche Diagnostic GmbH, Mannheim, Germany) (20)	10	NA	NA	NA	
	Procleix Ultrio Plus assay on the Tigris platform(Grifols Diagnostic Solutions, Emeryville, CA) (20)	3.4	NA	NA	NA	
	qPCR (52)	5	NA	NA	NA	Combined with a concentration of 2.5 mL plasma extraction volume, the LOD is 2 IU/ml (52).
	Nested PCR (52)	10	NA	NA	NA	Combined with a concentration of 2.5 mL plasma extraction volume, the LOD is 4 IU/ml (52).
	Procleix^®^ Panther^®^ system Procleix^®^ Ultrio Elite^®^ Assay (Novartis Diagnostics, Emeryville, CA, USA) (47)	ID-NAT: 4.3 (3.8–5.0) discriminatory HBV: 4.5 (4.0–5.3)	NA	NA	Only 42.03% donors were NAT repeated positive in the 138 repeat donors’ follow up tests. and the viral load level of them exhibit fluctuating state, which can affect the blood safety (47).	
	Procleix^®^ Tigris^®^ system Procleix^®^ Ultrio^®^ Assay (Novartis Diagnostics, Emeryville, CA, USA) (47)	ID-NAT: 10.4 (9.2–12.2) discriminatory HBV: 8.5 (7.6–9.8)	NA	NA		
	Procleix^®^ Tigris^®^ system Procleix^®^ Ultrio Plus^®^ Assay (Novartis Diagnostics, Emeryville, CA, USA) (47)	ID-NAT: 3.4 (3.0–4.1) discriminatory HBV: 4.1 (3.5–4.9)	NA	NA		
	CRISPR (122)	0.05 ng/ml	100%	NA	NA	
	NGS (127)	<2	>99.9%	NA	NA	

NA, not available

Although adoption of novel testing strategies has been shown to be effective in mitigating the transfusion-transmitted HBV infection, additional screening strategies may increase the financial burden on blood institutions and governments. Therefore, it is more cost-effective to optimize current screening protocols to establish a set of stable, reliable, and non-invasive detection paradigms for HBV markers to further ensure blood safety. Such as the HBsAg combined with HBV DNA ID-NAT screening protocol. Ye et al. (22). analyzed 132,323 donations using MP-6 HBV NAT and ID NAT comparison analysis, which showed that the yield of HBsAg-/DNA+ detected by ID NAT screening (0.12%) was 1.25 times that of MP NAT (0.058%, P < 0.05). To further enhance blood safety, HBsAg and HBV DNA ID NAT screening should be considered in regions/countries with high HBV prevalence. Besides, in low-resource areas, combined testing for HBsAg, anti-HBc, and anti-HBs can effectively reduce the risk of blood transfusion-transmitted HBV infection (129). Moreover, it is worth mentioning that anti-HBc screening and high-sensitivity HBV NAT screening can effectively prevent almost all HBV transmission from OBI donors (107, 109). To prevent HBV infection, it is recommended to perform ID-NAT on anti-HBc-positive blood donors, in order to discard plasma with weakly positive or negative anti-HBs but positive anti-HBc, or avoid transfusing anti-HBc-positive plasma to recipients with weakly positive or negative anti-HBs (130).

In summary, given the complexity of OBI, blood centers need adopt different strategies based on the risk of OBI transmission to improve blood safety. It is recommended that blood centers review the OBI risk assessment and identify OBI risk reduction strategies accordingly, including HBV vaccination, rigorous screening of blood donors, introduction of new technologies, exploration of OBI screening and shielding strategies, encouraging autologous transfusion and blood management, and pathogen inactivation of blood products from donors in areas with high prevalence of HBV.

## 8 Conclusions

Although the risk of transfusion-transmitted infection from blood components has been significantly reduced over the past few decades through rigorous donor selection and enhanced screening tests, a residual risk persists. TT-OBI remains the primary method of HBV transmission. Given the complexities associated with OBI and the absence of currently feasible screening and shielding strategies for blood centers in many countries, OBI presents significant challenges to blood safety. Therefore, it is essential to recruit low-risk voluntarily unpaid blood donors at the source; implement new technologies with mutation detection capacity to enhance sensitivity of current assays; develop appropriate screening and shielding strategies for donors who are OBIs; implement pathogen inactivation procedures, and optimize existing screening protocols to further prevent TT-OBI infections to ensure the highest possible level of blood safety.
